# Leptin Maintained Zinc Homeostasis Against Glutamate-Induced Excitotoxicity by Preventing Mitophagy-Mediated Mitochondrial Activation in HT22 Hippocampal Neuronal Cells

**DOI:** 10.3389/fneur.2018.00322

**Published:** 2018-05-09

**Authors:** Mei-fang Jin, Hong Ni, Li-li Li

**Affiliations:** Neurology Laboratory, Institute of Pediatric Research, Children’s Hospital of Soochow University, Suzhou, China

**Keywords:** leptin, zinc homeostasis, mitochondria, mitophagy, glutamate, HT22

## Abstract

Developmental seizure-induced long-term neuronal hyperexcitation is partially mediated by regenerative mossy fiber sprouting in hippocampus. Yet, there are no effective drugs available to block this pathological process. Recently, leptin has been shown to prevent the sprouting of hippocampal mossy fibers and abnormalities in the neurobehavioral parameters. However, their underlying molecular mechanisms are largely unknown. The purpose of this study was to determine the effect of glutamate on the parameters of zinc homeostasis, mitochondrial functions, and mitophagy regulating factors, as well as to investigate the protective effects of leptin against cytotoxicity of glutamate in murine HT22 hippocampal neuronal cells. Cells were assigned to one of the four groups as follows: control group, leptin alone group, glutamate injury group, and leptin pretreatment group. Our results demonstrated that glutamate induced a decrease in superoxide dismutase, GSH (glutathione), and mitochondrial membrane potential and an increase in GSSG (oxidized glutathione), mitochondrial reactive oxygen species, and supplementation of leptin blocked the toxic effect of glutamate on cell survival. The glutamate-induced cytotoxicity was associated with an increase in mitophagy and intracellular zinc ion levels. Furthermore, glutamate activated the mitophagy markers PINK1, Parkin, and the ratio of LC3-II/LC3-I, as well as increased the expression of zinc transporter 3 (ZnT3). Leptin corrected these glutamate-caused alterations. Finally, the mitophagy inhibitor, CsA, significantly reduced intracellular zinc ion content and ZnT3 expression. These results suggest that mitophagy-mediated zinc dyshomeostasis and mitochondrial activation contributed to glutamate-induced HT22 neuronal cell injury and that leptin treatment could counteract these detrimental effects, thus highlighting mitophagy-mediated zinc homeostasis *via* mitochondrial activation as a potential strategy to counteract neuroexcitotoxicity.

## Introduction

Leptin, a 16-kDa peptide hormone produced by white adipocytes, was cloned from the ob gene in 1994 as an important milestone in reducing obesity (1). From then on, leptin targets have been widely found in the brain. Leptin acts as a pleiotropic hormone in neuronal morphology, activity-dependent synaptic plasticity, and cognition (2, 3). Leptin has recently been discovered to exert neurotrophic effects and neuroprotective activity, which can slow down the process of neuronal damage after acute brain injury as well as during long-term neurodegenerative processes (4). Acute leptin application could protect neurons against cell death induced by excitotoxic and oxidative insults in hippocampus (5). Platt et al. reported that leptin-resistant mice had elevated levels of tau phosphorylation, while cells cultured with leptin reduced tau phosphorylation (6). Using a mouse model of traumatic brain injury (TBI), Lopez-Rodriguez et al. recently demonstrated that leptin (administered once, i.p. immediately after lesion) recovered several parameters affected by TBI, including the expression of cannabinoid receptors, axonal injury marker, and neuroinflammatory components (7). Meanwhile, Malekizadeh et al. further showed that the leptin (116–130) fragment mirrored the ability of leptin to facilitate activity-dependent hippocampal synaptic plasticity, promote the cognitive enhancing effects of leptin, and prevent hippocampal synaptic disruption and neuronal cell death (8). Erbayat-Altay et al. found that leptin-deficient ob/ob mice increased the severity of pentylenetetrazol-induced seizures (9). Studies have also shown that leptin has neuroprotective effects on the status epilepticus (SE) induced by kainic acid or pilocarpine (10, 11).

Among the molecular mechanisms responsible for the effects of leptin, pro-survival signaling appears to play a major role (12). Recently, we developed a “double hit” seizure model that initially used pilocarpine to induce SE in the neonatal period and then used penicillin to induce a second SE in adolescence or adulthood. The results showed that leptin treatment prevented long-term abnormalities in cognition, seizure threshold, hippocampal mossy fiber sprouting, and zinc transporter 3 (ZnT3)/CB-D28k expression (13). This highlights ZnT3/CB-D28k-associated Zn (2+)/Ca (2+) signaling as a promising therapeutic target. Zinc has activity-dependent regulation of neuronal circuits beyond hippocampal mossy fibers to excitatory fibers (14). The ZnT3-dependent “zincergic” vesicular zinc accounts for hippocampal synaptic vesicle zinc levels and is associated with mossy fiber sprouting and cognitive deficits (15, 16). Our previous study also demonstrated that the expression of zinc transporters ZnT-3, MT-3, and lipid metabolism-related molecules ApoE and clusterin, together with ACAT-1, a mitochondrial enzyme that catalyzes the esterification of cholesterol and long-chain fatty acid to form a cholesterol ester, were downregulated by a ketogenic diet (KD) following neonatal seizures, in parallel with mossy fiber sprouting in hippocampus and deficits of cognition. This suggests that zinc-induced lipid peroxidation and mitochondrial metabolic pathways may be involved in the target of KD (17). Intervention studies with a mitochondrial targeting molecule melatonin also restored the long-term hippocampal mossy fiber sprouting and neurobehavioral changes following recurrent neonatal seizures, which may be associated with ACAT-1/cathepsin-E signaling (18, 19). In another study using the same neonatal seizure model, we found that chronic KD treatment restored the upregulation of clusterin and the autophagy markers beclin-1, p62, and cathepsin-E, indicating that the abovementioned zinc/mitochondrial metabolic pathway may be involved in autophagy mechanisms (20). As previous gene chip studies have shown, cathepsin E is the target of the neuroprotective effects of the KD (21). Together with the fact that cathepsin E is an executive member of autophagy, it is reasonable to speculate that the long-term anticonvulsant or neuroprotective effects of leptin may be mediated by zinc and autophagy signals.

Thus, we hypothesized that the mitophagy pathway, together with zinc/mitochondrial homeostasis signaling, may be involved in the protective effect of leptin against excitotoxic neuronal damage. In this respect, we used an *in vitro* model of glutamate-induced excitotoxicity injury in hippocampal HT22 cells. Cell viability, the parameters of mitochondrial function and zinc homeostasis, and the biomarkers for mitophagy were measured.

## Materials and Methods

### Cell Lines and Culture Conditions

HT22 Mouse Hippocampal Neuronal Cell Line was purchased from the Institute of Biochemistry and Cell Biology of the Chinese Academy of Sciences (Shanghai, China). All cells were cultured in DMEM (GIBCO-BRL) supplemented with 10% fetal bovine serum (Serana, Germany), 100 U/ml of penicillin, and 100 mg/ml of streptomycin (Beyotime) in humidified air at 37°C with 5% CO_2_. Cells were seeded into each well of a 6-well plate or 96-well plate the day before the experiment. After treatments, HT22 cells were subjected to various measurements as described below.

### Reagents and Antibodies

l-glutamate and cylosporinA were obtained from Sigma-Aldrich (St. Louis., MO, USA). Recombinant murine leptin was purchased from PeproTech (Rocky Hill, NJ, USA), and protein molecular weight marker was purchased from Thermo Fisher Scientific (Waltham, MA, USA). RIPA lysis buffer and a BCA Protein Assay Kit were purchased from Beyotime Institute of Biotechnology (Shanghai, China). Primary antibodies against PINK1, Parkin, and LC3 were obtained from Abcam (Cambridge, MA, USA). Antibodies against β-actin were obtained from Sigma-Aldrich (St. Louis., MO, USA), and antibodies against ZnT3 were obtained from Santa Cruz Biotechnology (Dallas, TX, USA). The secondary antibodies for immunoblots were HRP-conjugated anti-rabbit, anti-mouse, and anti-goat IgG (Beyotime, Shanghai, China).

### Cell Viability Assay

Cell viability assays were performed using Cell Counting Kit-8 (Dojindo Molecular Technologies, Kumamoto, Japan) following the manufacturer’s protocol with cultured cells (at a concentration of 5 × 10^4^) in a 96-well plate at a volume of 100 μL/well. After treatment, 10 µl of Cell Proliferation Reagent CCK8 was added to each well and incubated for 3 h at 37°C and 5% CO_2_. For background control, 100 µl of culture medium and 10 µl of Cell Proliferation Reagent CCK8 were added to a single well. The mixtures were shaken for 1 min using a shaker, and the absorbance of the samples was measured at 450 nm using a microplate reader. The experiment was performed in triplicate for each group and repeated three times. Cell survival probability was calculated by comparison with the control group.

### Lactate Dehydrogenase (LDH) Assay

The LDH release assay was used to determine the membrane integrity, as intracellular LDH would release into the extracellular media if the cellular membrane was damaged. LDH concentration in the culture medium was measured using a diagnostic kit (Nanjing Jiancheng Bioengineering Institute, Nanjing, China) according to the manufacturer’s instructions. Cells (at a concentration of 5 × 10^4^) were cultured in a 96-well plate at a volume of 100 µl per well. After treatment, the supernatant was obtained by centrifugation (12,000 rpm for 3 min), and 50 µl of supernatant was seeded in a new 96-well plate and then incubated with the reduced form of nicotinamide-adenine dinucleotide (NADH) and pyruvate for 15 min at 37°C. The reaction was stopped by adding 0.4 mol/l of NaOH. Activity of LDH was calculated from absorbance at 440 nm, and the background absorbance from the culture medium that was not used for cell culture was subtracted from all absorbance measurements (22, 23).

### Biochemical Analysis of Oxidative Stress Markers

After treatments, the cells in 6-well plate were washed three times with pre-cooling PBS and then lysed in RIPA buffer containing phosphatase inhibitor mixture tablets and protease inhibitor mixture tablets (Roche Applied Science, Mannheim, Germany). The supernatant was collected and protein concentration was determined using a BCA protein assay kit (Beyotime, Shanghai, China). Superoxide dismutase (SOD), GSH, and GSSG levels were measured using a SOD, GSH, and GSSG Assay Kit (Nanjing Jiancheng Bioengineering Institute, Nanjing, China), performed according to the manufacturer’s instruction (24).

### Analysis of Mitochondrial Membrane Potentials

The ΔΨm was determined using the dual-emission mitochondrion-specific lipophilic, JC-1 (Beyotime, Shanghai, China), a mitochondrial potential indicator that exists either as a green fluorescent monomer at depolarized membrane potentials or as a red fluorescent J-aggregate at hyperpolarized membrane potentials. The cells were harvested from experimental samples, and the total volume was brought to 0.5 ml of fresh complete medium. Cell suspensions were stained with 0.5 ml of JC-1 working solution. Each tube was gently vortexed and kept in a dark place at room temperature for 15–20 min. Samples were washed twice, centrifuging at 500 × *g* for 5 min with a double volume of incubation buffer. Next, samples were resuspended in 0.3 ml of incubation buffer, and the green fluorescent JC-1 monomers and the red fluorescent J-aggregates in the cells were detected with a Gallios Flow Cytometer (Beckman Coulter, Brea, CA, USA). Green fluorescence was detected through the FL1 channel and red fluorescence through the FL2 channel. The data were analyzed by using the FlowJo Analysis Software and displayed in a dot plot of J-aggregate red fluorescence (*y*-axis) against JC-1 green fluorescence (*x*-axis) (25, 26).

### Determination of Mitochondria Reactive Oxygen Species (ROS) (MitoSOX)

MitoSOX™ (Thermo Fisher Scientific, Waltham, MA, USA), a red mitochondrial superoxide indicator, is a novel fluorogenic dye for highly selective detection of superoxide in the mitochondria of live cells. After various treatments, the medium was removed, and the cells were washed with prewarmed HBSS. MitoSOX reagent working solution (1.0 ml of 5 µM) was applied to cover cells. Cells were incubated for 10 min at 37°C and protected from light. Cells were then washed gently three times with HBSS. Nuclei were stained with 1 µg/ml of blue-fluorescent Hoechst 33342 for 10 min, and cells were washed gently three times with HBSS. Cells were counterstained as desired and mounted in warm buffer for imaging.

### Mitophagy Assay

A mitophagy detection kit (Dojindo Molecular Technologies, Kumamoto, Japan) was used to monitor mitophagy in HT22 cells. This kit is composed of LysoTracker Red and MitoTracker Green Dye. HT22 cells were seeded in confocal dished and cultured overnight. Cells were washed twice Hanks’ HEPES buffer and then incubated with Mtphagy Dye for 30 min. After washing again cells were treated with glutamate and/or leptin. After incubation for 24 h, removing the supernatant and washing the cells, then added Lyso Dye to the cells and incubated for 30 min. Then, the cells were washed twice, and the Mtphagy and Lyso Dye were observed using a confocal fluorescence microscopy.

### Western Blot Assay

Mitochondrial proteins were extracted using a Mitochondria Protein Extraction Kit (EnoGene, Nanjing, China). Cells were collected after treatment, rinsed, and lysed in RIPA buffer containing protease and phosphatase inhibitor cocktails. After centrifugation at various speeds, the cytosol and nucleus were isolated from subcellular fractions. The cytosol fraction was lysed in mitochondria protein extraction buffer. After centrifugation at various speeds, mitochondria protein was isolated (27). Mitochondria proteins were separated by SDS-PAGE, transferred to a PVDF membrane, and probed with β-Actin (1:1,000), PINK1 (1:1,000), Parkin (1:1,000), LC3 (1:1,000), and ZnT3 antibodies. Horse-radish peroxidase conjugated anti-rabbit IgG was used as a secondary antibody (1:1,000). The detection was performed using the Pierce™ Western Blotting Substrate (Thermo Scientific Pierce), and then the blots were analyzed by ImageJ for their integrated density.

### Measurement of Intracellular Zinc Ions Concentration

Zinquin ethyl ester (Dojindo Molecular Technologies, Kumamoto, Japan) is membrane permeable and, therefore, useful in detecting intracellular zinc ions. The supernatant was discarded after treatment, washed with prewarmed HBSS Buffer twice, and then 250 µl of 12 µM Zinquin ethyl ester was added to the cells and incubated at 37°C for 30 min. The cells were washed three times with HBSS buffer and then observed using a confocal fluorescence microscopy.

### Determination of Intracellular Zinc Ions Concentration of CsA-Incubated Cells

CyclosporinA (CsA) (mitophagy inhibitor) was used to explore the biological effects of mitophagy on glutamate-induced zinc dysfunction. The HT22 cells were divided into two groups. The Glutamate group was cultured with 5 mmol/l of glutamate, and the CsA intervention group was given CsA (0.5 µmol/l) for 2 h prior to glutamate treatment. Mitophagy was detected with a mitophagy detection kit and Western blot. Zinquin ethyl ester and Western blot were used to measure intracellular zinc ions and ZnT3, respectively.

### Statistical Analysis

The data are presented as the mean ± SEM from three independent experiments with three replicates evaluated for each experiment. Statistical analysis was done using GraphPad Prism version 5.0 (La Jolla, CA, USA). The statistically significant differences between groups were assessed by analysis of variance followed by a Bonferroni *post hoc* test when more than two groups or by an unpaired Student’s *t*-test for two samples. A *P*-value of <0.05 was considered significant.

## Results

### Leptin Attenuates Glutamate-Induced Excitotoxicity in HT22 Cells

**Figure 1:**
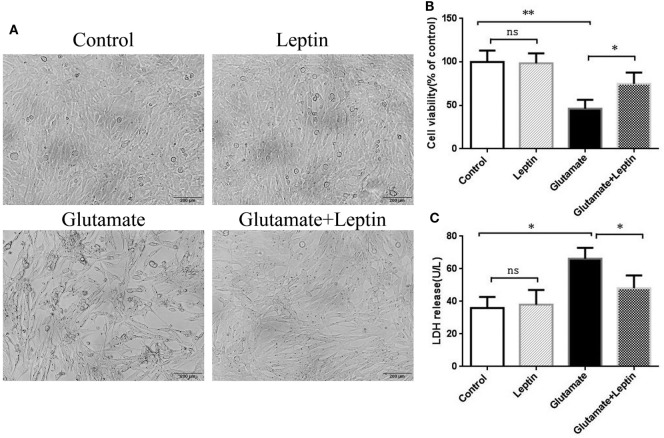
Neuroprotective effects of leptin against glutamate-induced excitotoxicity. (**A)** The morphology of HT22 cells were visualized under a light microscope; **(B)** cell viability was measured by CCK8 assay; **(C)** neurotoxicity was measured by lactate dehydrogenase assay. *P < 0.05, **P < 0.01; ns, not significant.

To explore the biological effects of leptin on glutamate-induced excitotoxicity, hippocampal neuronal cells HT22 were pretreated with leptin before glutamate injury. Based on the relevant literature (28) and combined with our preliminary experiments, we used different concentrations of leptin (10, 100 ng/ml, 1, 10 µg/ml) for efficacy tests. We found that 1 µg/ml leptin can significantly increase cell survival (at 5 mmol/ml glutamate). We further examined the effect of leptin alone at a concentration of 1 µg/ml on cell survival and found that this concentration had no effect on cell survival, demonstrating that this concentration has no cytotoxic effects on the cells. Accordingly, in this study, l μg/ml leptin concentrations were used for experimental studies. HT22 cells were studied in four groups: (1) Control group (Control), (2) Leptin alone group (Leptin) (leptin (1 µg/ml) was added to cells), (3) glutamate injury group (Glutamate) (the glutamate was added to the culture medium to obtain a final concentration at 5 mM), and (4) leptin pretreatment group (Glutamate + Leptin) (cells were pretreated with 1 µg/ml leptin culture for 2 h, and then given glutamate to the culture medium to obtain a final concentration at 5 mM). Cells were further incubated at 37°C, 5% CO_2_ for 24 h. The results showed that glutamate exposure caused cell shrinkage and nuclear condensation in HT22 cells, indicative of cell death (Figure 1A). Leptin preserved cell density and restored normal morphology features. Consistent with the morphology results, the viability of HT22 cells was higher than that of HT22 cells without leptin pretreatment with the same concentration of glutamate (Figure 1B). Furthermore, leptin pretreatment decreased LDH release after glutamate treatment (Figure 1C). Thus, leptin significantly reversed the glutamate-induced decrease of HT22 cell viability and the increase in LDH release. In addition, a single administration of leptin did not cause injury to cells, indicating that leptin exerted a protective role on glutamate-induced impairment of HT22 cells.

### Protection of Leptin Against Glutamate-Induced Oxidative Stress in HT22 Cells

**Figure 2:**
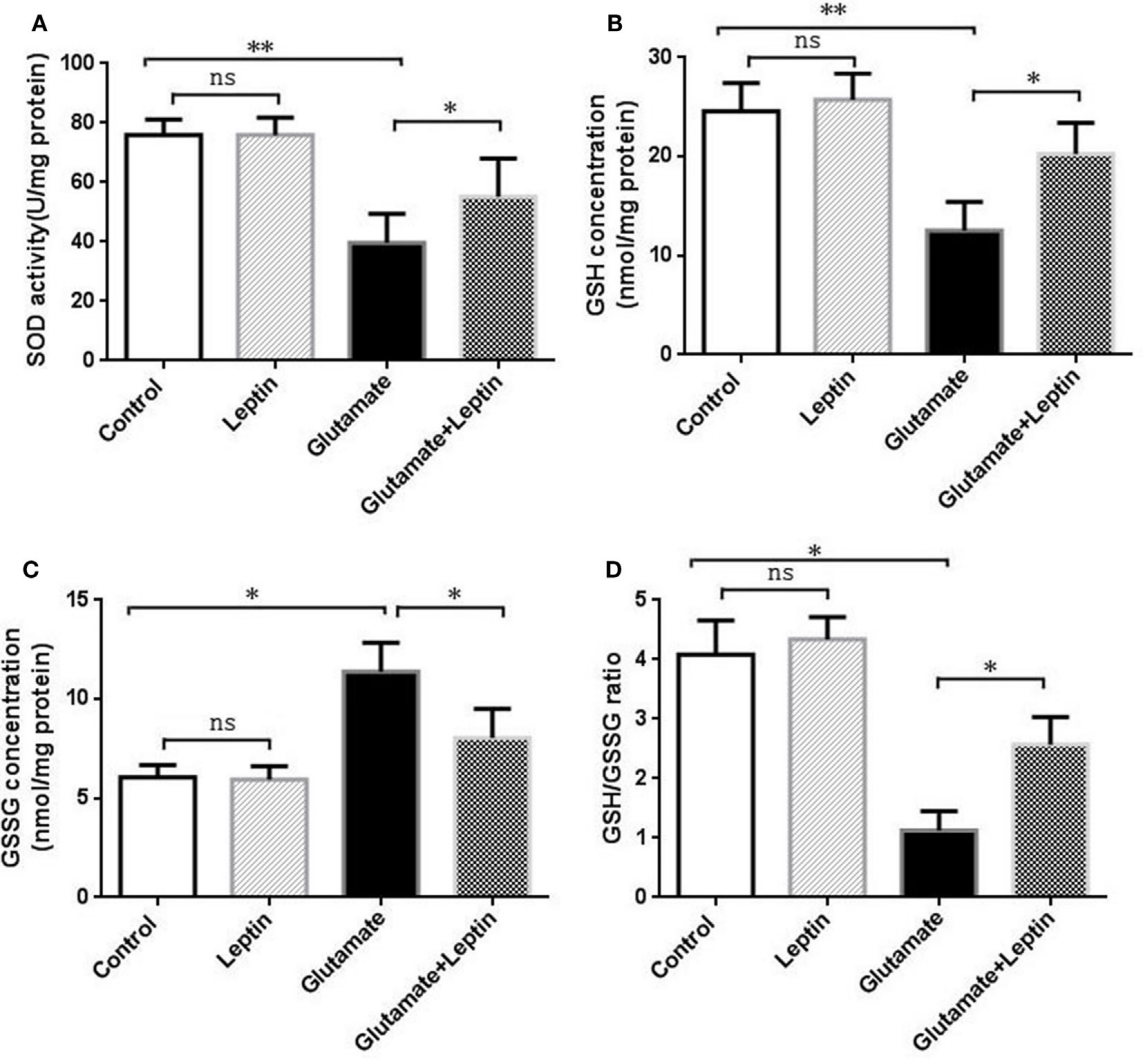
Antioxidant function of leptin against glutamate-induced oxidative stress in HT22 cells. **(A)** The effect of leptin on superoxide dismutase activity; **(B)** the effect of leptin on GSH concentration; **(C)** the effect of leptin on GSSG concentration; **(D)** the effect of leptin on the GSH/GSSG ratio. *P < 0.05, **P < 0.01. ns, not significant.

To evaluate whether the neuroprotection provided by leptin was associated with its antioxidative activities, indicators of oxidative stress were measured, including SOD, GSH, GSSG, and GSH/GSSG. Cells were treated according to the above methods, and then the oxidative stress assays were performed according to the manufacturer’s instructions. Data analysis revealed that glutamate could significantly decrease SOD activity, GSH concentration, and the GSH/GSSG ratio compared to the Control group (Figures 2A,B,D). Meanwhile, glutamate could significantly increase GSSG concentrations compared with the Control group (Figure 2C). Moreover, leptin could significantly reverse the changes in oxidative indicators triggered by glutamate compared to the glutamate injury group, while a single usage of leptin did not exert any influence at the cellular oxidative level, suggesting that the protective effects of leptin may be mediated *via* its antioxidant function.

### Leptin Prevents Glutamate-Induced Mitochondrial Dysfunction and Mitochondrial ROS Accumulation

Since oxidative stress is usually associated with the energy state of the mitochondria (mitochondrial membrane potential) and the aggregation of ROS, we further explored the effect of leptin on the mitochondrial membrane potential and mitochondrial ROS changes induced by glutamate.

**Figure 3:**
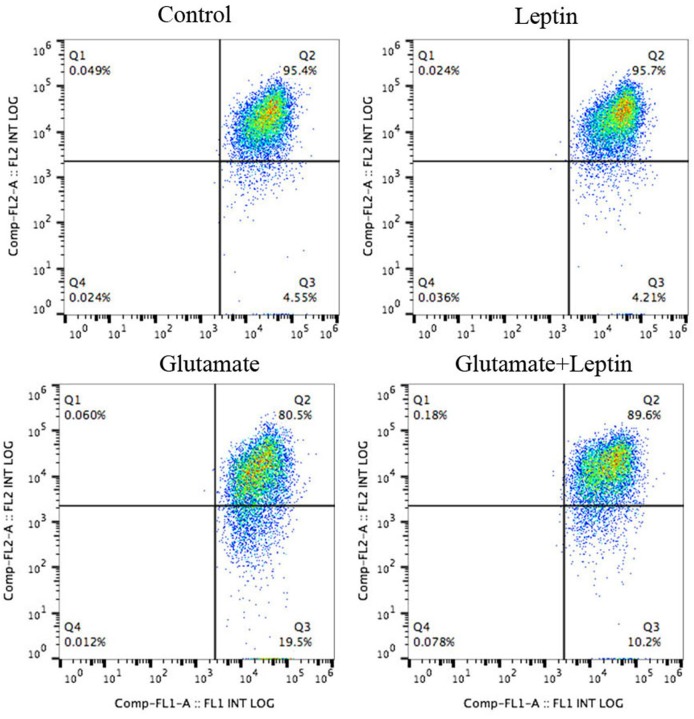
Leptin preserves glutamate-induced mitochondrial membrane depolarization.

Thus, cells were treated according to the above methods, and after 24 h of treatment, the mitochondrial membrane potential (ΔΨm) was measured by JC-1 (Figure 3). Flow cytometry was used to detect the fluorescence ratio of the FL-1 channel (green fluorescence) and the FL-2 channel (red fluorescence). As a result, after glutamate treatment, the green fluorescent cells gradually increased, indicating increasing cells with mitochondrial transmembrane potential depolarization. Leptin intervention could significantly reverse the migration of cell populations and increase the mitochondrial membrane potential in cells, implicating the important role of leptin in maintaining the normal level of mitochondrial membrane potential.

**Figure 4:**
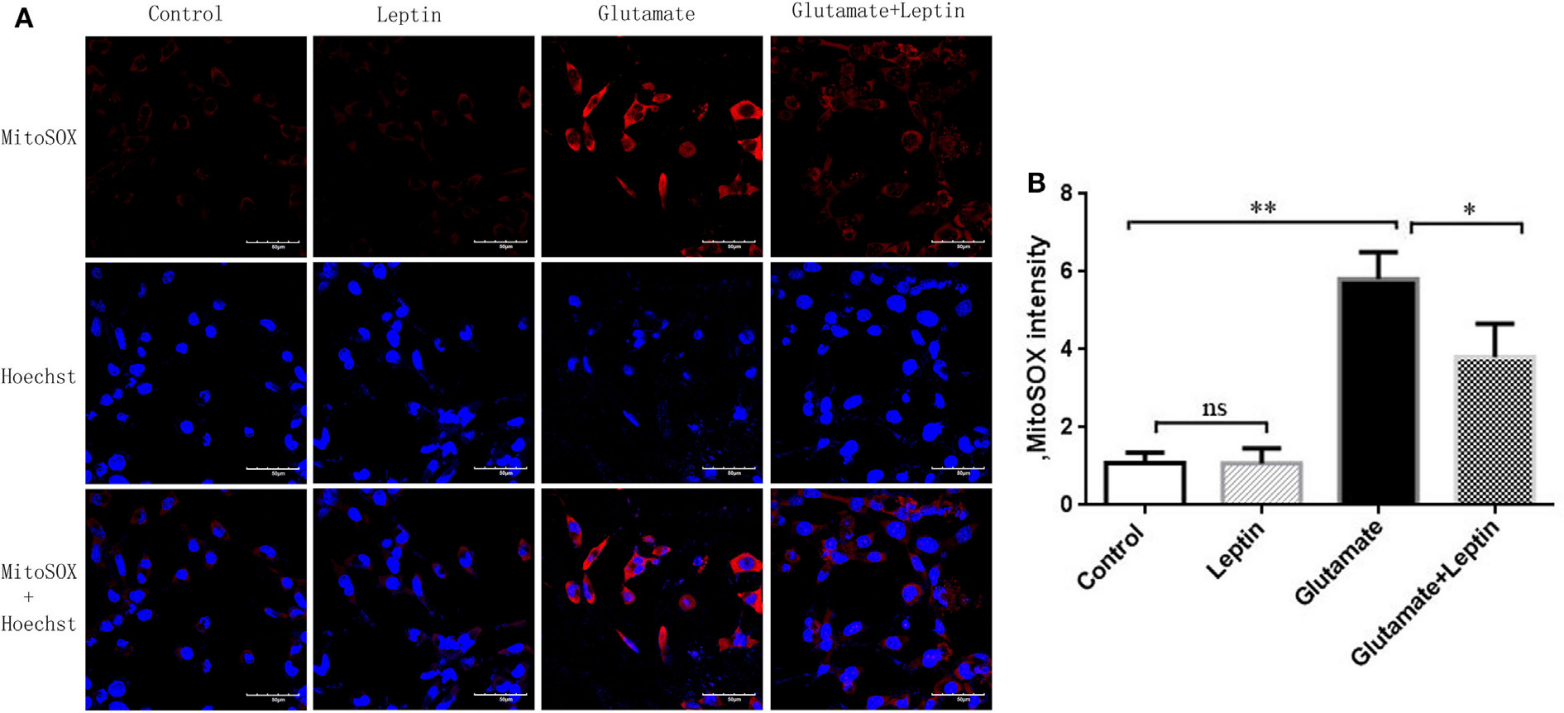
Leptin reduces mitochondrial reactive oxygen species (ROS) accumulation. **(A)** The fluorescence intensity of mitochondrial ROS in HT22 cells; **(B)** the quantitative analysis of average fluorescence intensity of mitochondrial ROS. *P < 0.05, **P < 0.01. ns, not significant.

As mitochondrial dysfunction was associated with an excess of mitochondrial ROS, we next detected mitochondrial ROS with a MitoSOX probe (Figure 4A). The MitoSOX probe demonstrated (Figure 4B) that mitochondrial ROS was significantly increased in HT22 cells after 24 h administration with 5 mM of glutamate compared to the Control group, demonstrating that glutamate promoted the generation of ROS in mitochondria of HT22 cells. Leptin pretreatment could significantly reduce mitochondrial ROS accumulation induced by glutamate. However, a single addition of leptin did not affect the mitochondrial ROS content, suggesting the leptin exerted a protective role in HT22 cells by inhibiting the ROS accumulation triggered by glutamate.

### Leptin Inhibited Glutamate-Induced Mitophagy and Zinc Metabolism Disorder

**Figure 5:**
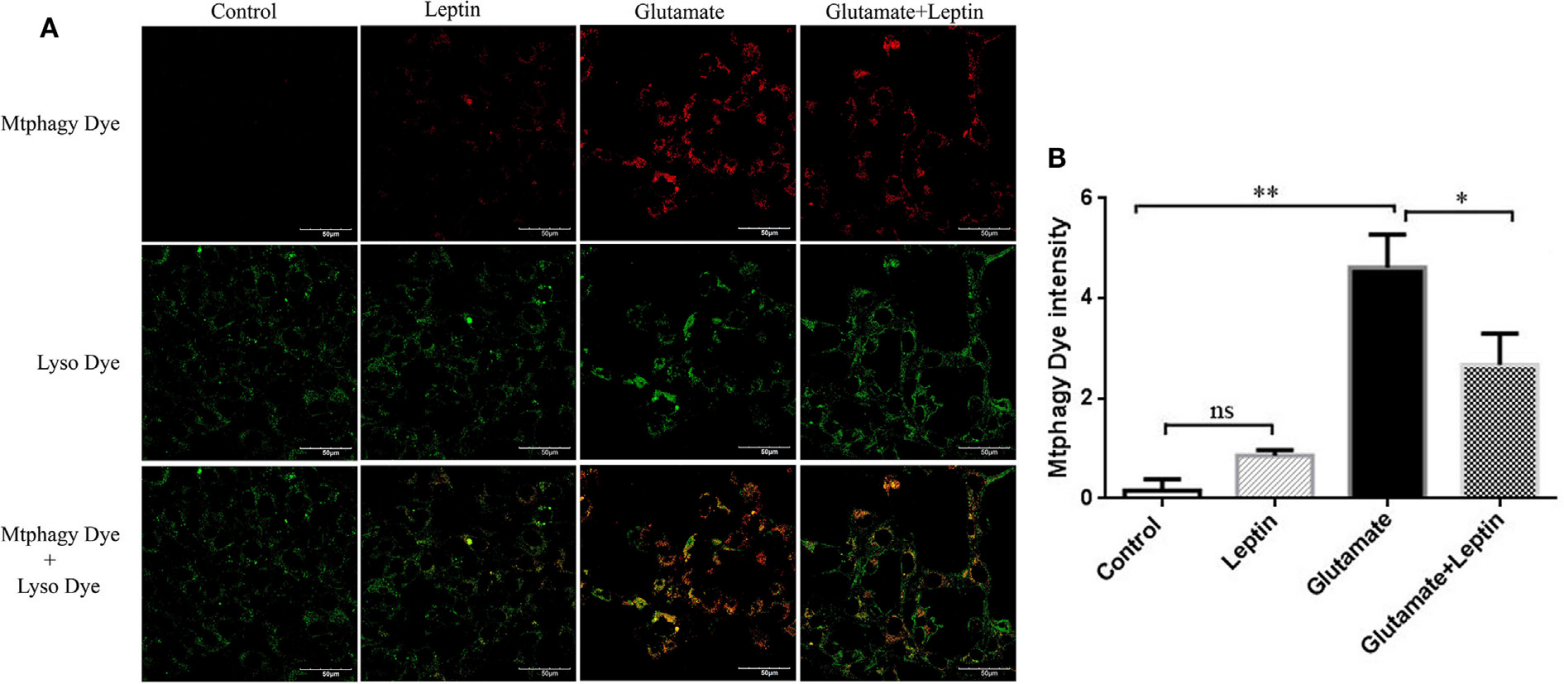
The effect of leptin on mitophagy induced by glutamate in HT22 cells. **(A)** The fluorescence intensity of Mtphagy Dye and Lyso Dye in mitochondria of HT22 cells; **(B)** the quantitative analysis of the average fluorescence intensity of mitochondrial Mtphagy Dye in HT22 cells. *P < 0.05, **P < 0.01. ns, not significant.

A mitophagy detection kit was used to detect the effect of leptin on glutamate-induced abnormalities of mitophagy. Exposure of HT22 cells to 5 mM of glutamate for 24 h led to an increase in Mtphagy Dye fluorescence intensity, and leptin pretreatment (1 µg/ml) significantly decreased the fluorescence intensity of Mtphagy Dye, indicating that leptin could suppress the upregulation of mitophagy induced by glutamate. Single administration of leptin upregulated the fluorescence intensity of Mtphagy Dye in HT22 cells to some extent, though without statistical significance (Figures 5A,B).

**Figure 6:**
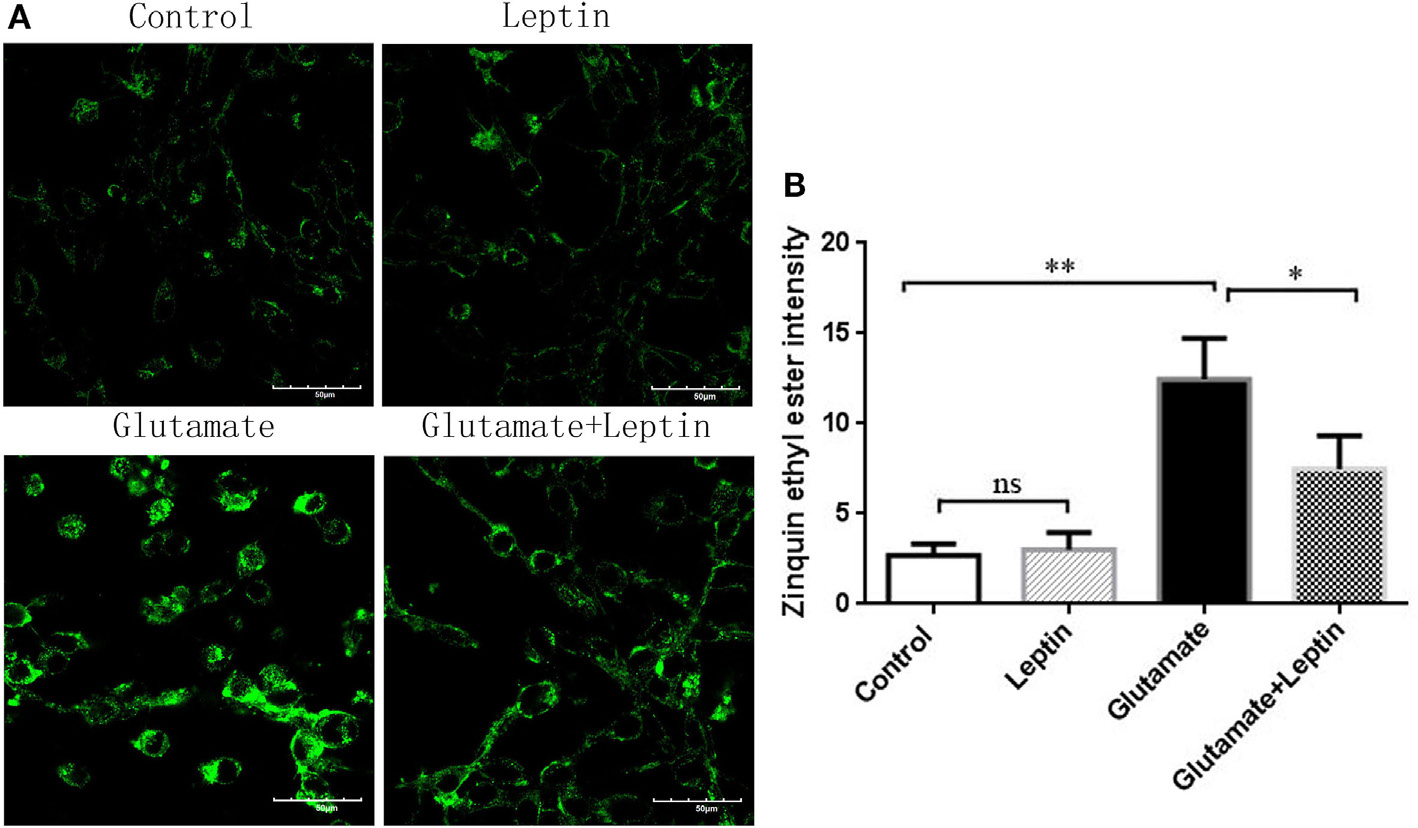
The effect of leptin on zinc ion level in glutamate-treated HT22 cells. **(A)** The fluorescence intensity of Zinquin ethyl ester in HT22 cells; **(B)** the quantitative analysis of the average fluorescence intensity of Zinquin ethyl ester in HT22 cells. *P < 0.05, **P < 0.01, ns, not significant.

At the same time, we used a zinc ion fluorescence probe to detect the concentration of intracellular free zinc ions. The cells were treated according to the above methods, and our result showed that glutamate administration led to a significant increase in the fluorescence intensity of Zinquin ethyl ester, a fluorescent probe of zinc ion in HT22 cells, while leptin pretreatment significantly decreased the cellular zinc ion level. This indicates that leptin can inhibit glutamate-induced upregulation of zinc ion in HT22 cells (Figures 6A,B). The zinc ion fluorescence probe stain illustrated the free zinc ion concentrations had a positive significant association with the mitophagy level.

**Figure 7:**
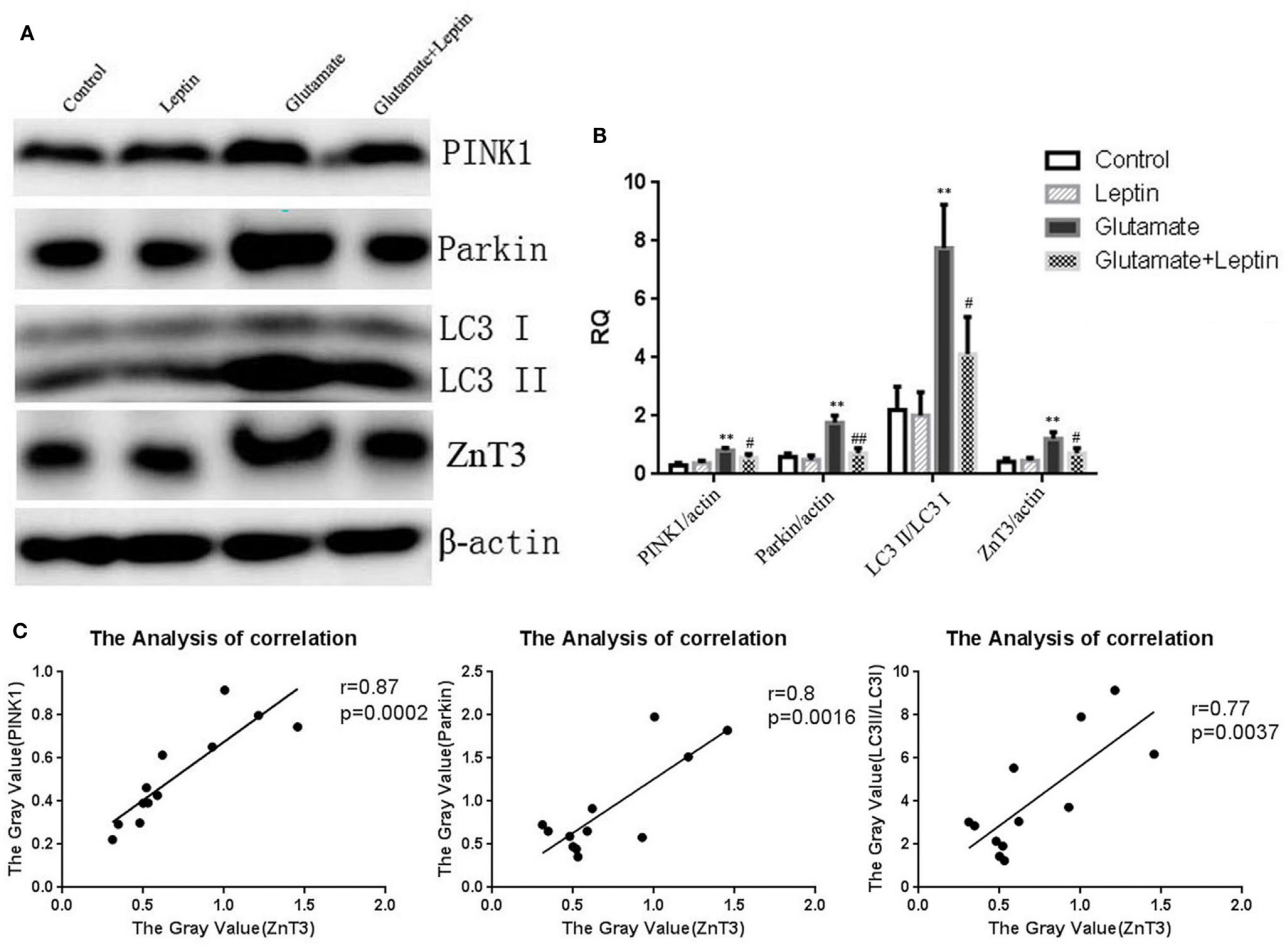
The effect of leptin on glutamate-triggered expressions of mitophagy protein and zinc transporter 3 (ZnT3). **(A)** Western blot assay of expressions of mitochondrial autophagy protein and ZnT3 in HT22 cells; **(B)** the relative expression (integral optical density) of mitochondrial autophagy protein and ZnT3 in HT22 cells; **(C)** the analysis correlation between ZnT3 and mitophagy-related proteins *P < 0.05, **P < 0.01, compared to the control group; ^#^*P* < 0.05, ^##^*P* < 0.01, compared to the Glutamate group.

To further clarify the effect of leptin on mitophagy and zinc metabolism, Western blot was used to detect the expression of mitophagy related protein and ZnT3. Immunoblot analysis showed that incubation of cultures with 5 mM of glutamate for 24 h resulted in highly significant increases in the expression of PINK1, Parkin, and the LC3 II/I ratio. ZnT3 expression was significantly upregulated as well, and 1 µg/ml of leptin pretreatment could reverse this change (Figures 7A,B). This suggests that leptin could simultaneously suppress the glutamate-induced upregulation of mitophagy and ZnT3. Therefore, we further analyzed the correlation between ZnT3 and mitophagy-related proteins. The results showed that the expression of ZnT3 was positively correlated with the expression of mitophagy proteins Parkin, PINK1, and LC3 II/I (Figure 7C).

### Leptin Regulates the Homeostasis of Zinc Ions Through Mitochondrial Autophagy Pathway

**Figure 8:**
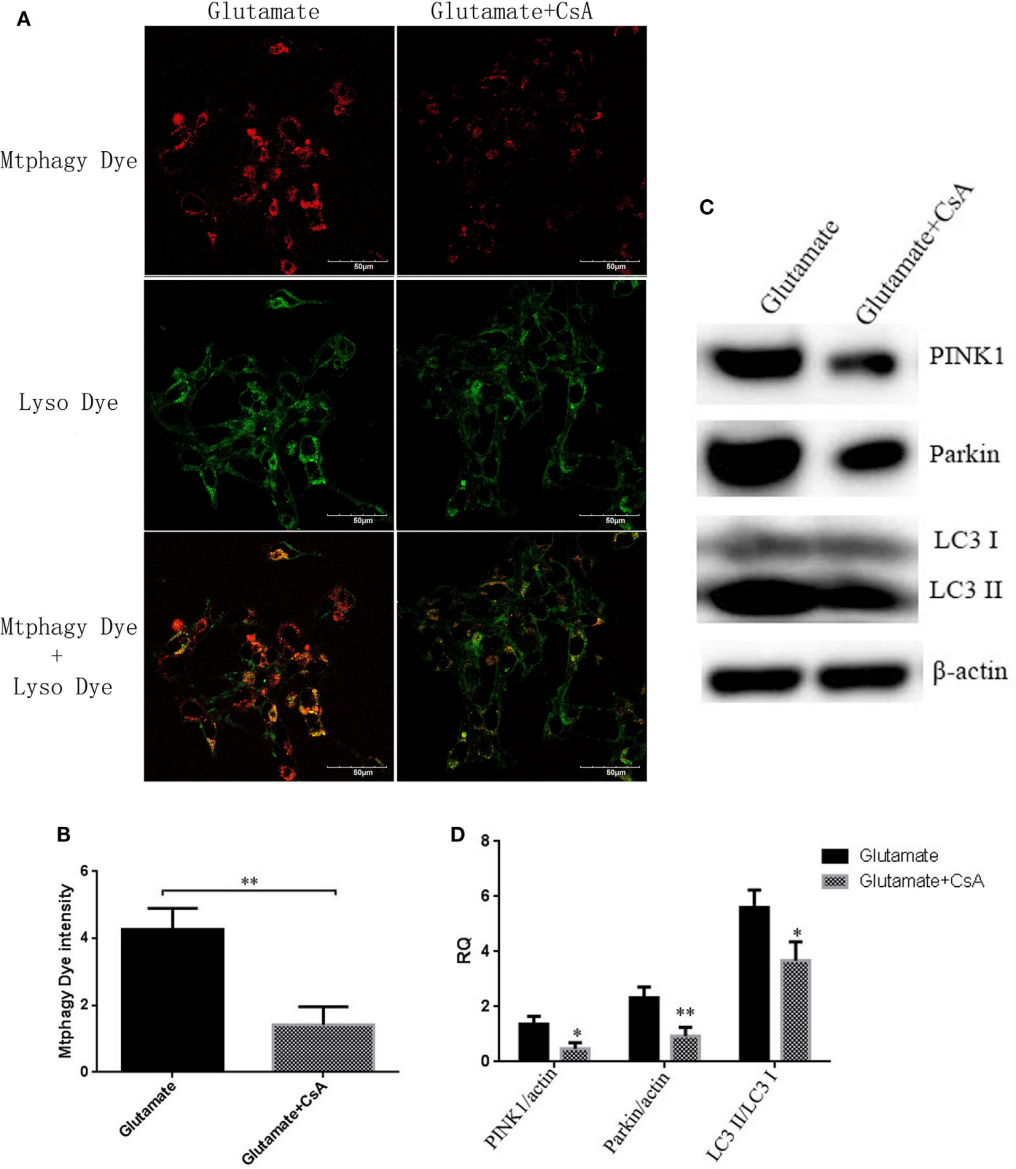
The effect of cyclosporinA (CsA) on glutamate-triggered mitochondrial autophagy. **(A)** The fluorescence intensity of Mtphagy Dye and Lyso Dye in mitochondria of HT22 cells; **(B)** the quantitative analysis of the average fluorescence intensity of mitochondrial Mtphagy Dye in HT22 cells; **(C)** Western blot assay of mitochondrial autophagy protein in HT22 cell; **(D)** the relative expression (integral optical density) of mitochondrial autophagy protein in HT22 cells. *P < 0.05, **P < 0.01. ns, not significant.

**Figure 9:**
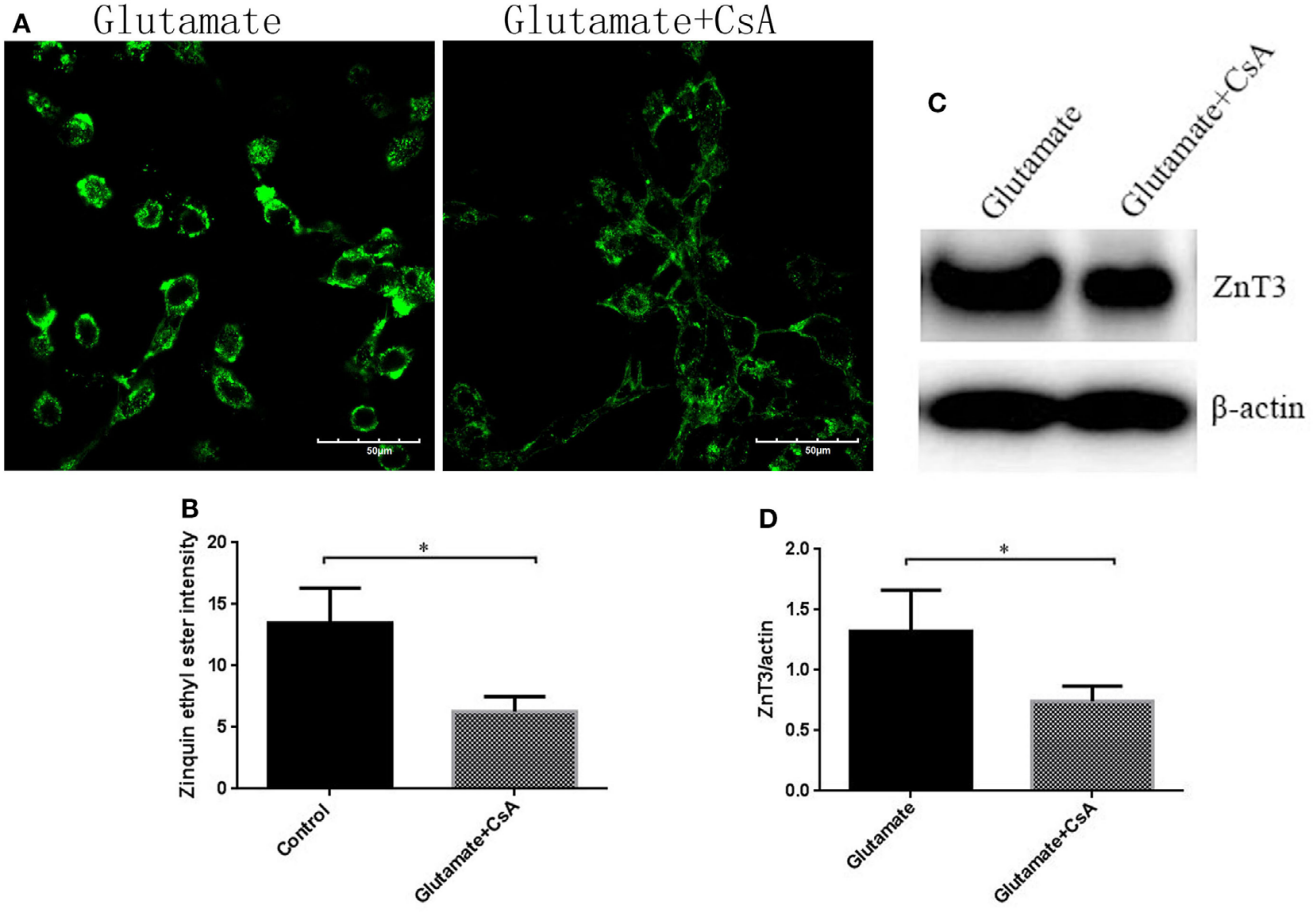
The effect of cyclosporinA (CsA) on glutamate-triggered cellular zinc disorder. **(A)** The fluorescence intensity of Zinquin ethyl ester in HT22 cells; **(B)** the quantitative analysis of the average fluorescence intensity of Zinquin ethyl ester in HT22 cells; **(C)** Western blot assay of zinc transporter 3 (ZnT3) in HT22 cell; **(D)** the relative expression (integral optical density) of ZnT3 in HT22 cells. *P < 0.05, **P < 0.01. ns, not significant.

To further determine whether the regulatory function of leptin on the metabolism of zinc ions was mediated by mitophagy in HT22 cells, we used CsA, an inhibitor of mitophagy, to observe the effect of mitophagy on cellular zinc ion and zinc ion transporter. CsA is able to inhibit the reduction of membrane potential and simultaneously suppress the occurrence of mitophagy. After incubating for 24 h, CsA was effective in reducing the fluorescence intensity of Mtphagy Dye (Figures 8A,B) and the expression of mitophagy proteins (Figures 8C,D), simultaneously leading to a significant reduction in cellular zinc ion content (Figures 9A,B), as well as the expression of zinc ion transporter (Figures 9C,D). These findings implicated that mitophagy was involved in the zinc metabolism pathway.

## Discussion

In present study, the effects of adipokine leptin and the signaling pathway involved were investigated in glutamate-exposed HT22 cells. There are two key findings of this study. First, glutamate resulted in decreased concentrations of SOD and glutathione (GSH), reduced mitochondrial membrane potential, and increased the content of oxidized glutathione (GSSG), mitochondrial ROS. In addition, glutamate induced the increased activity of mitochondrial autophagy (mitophagy), increased levels of intracellular zinc ion and ZnT3, and upregulated the expression of mitophagy markers PINK1, Parkin, and the LC3-II/LC3-ratio. Leptin corrected these glutamate-caused alterations. Second, the mitophagy inhibitor, CsA, significantly reduced the intracellular zinc ion content and ZnT3 expression. These results suggest mitophagy mediated-zinc dyshomeostasis and mitochondrial dysfunction contributed to glutamate-induced HT22 neuronal cell injury, and leptin treatment could counteract these detrimental effects.

Sustained high levels of extracellular glutamate are a major cause of excitotoxicity during seizures. Jayaram et al. found that glutamate increased *in vitro* ObRa and ObRb expression, whereas leptin pretreatment attenuated glial cytotoxicity by excess glutamate, reflected by better preserved adenosine triphosphate production (11). This is in accordance with our present study. Herein, we revealed that leptin treatment counteracted glutamate-induced cytotoxicity. However, our research differs from that of Jayaram in the following aspects. In Jayaram’s study, C6 astrocytoma cells were used. However, in our study, the cell type was HT22 hippocampal neurons. In addition, Jayaram et al. mainly investigated the expression of ObRa and ObRb and ATP levels. However, in the present study, we focused on parameters of mitochondrial function and zinc homeostasis, as well as the biomarkers for mitophagy, especially the interaction between mitophagy and zinc homeostasis signaling.

Previous studies have demonstrated that zinc signaling is involved in the elevated expression of lipid metabolism related proteins, such as ApoE, clusterin, and cholesterol, which might participate in the pathological process of AD (29, 30). We previously showed that chronic KD treatment restored the long-term increased expression of ZnT-3, MT-3, and ApoE/ApoJ in hippocampus following neonatal seizure attacks (17), which paralleled hippocampal mossy fiber sprouting and cognitive abnormalities as previously reported (31, 32). These studies highlight the possibility that zinc-related lipid peroxidation might be a potential target for the treatment of seizure-induced brain injury. The current *in vitro* study provides evidence to support this hypothesis. Here, compared with the control, glutamate-treated neuronal cells showed elevated lipid peroxidation-related indicators, including SOD, reduced glutathione (GSH), and oxidized glutathione (GSSG), as well as decreased mitochondrial ROS activity and mitochondrial membrane potential. In addition, supplementation of leptin prevented these detrimental effects of glutamate on cell survival.

Here, we further investigated the underlying molecular mechanisms of the toxic effect of glutamate on neuronal cells by measuring the protein levels of mitophagy and zinc homeostasis signaling. We showed that glutamate induced an increase in mitochondrial autophagy, intracellular zinc, and ZnT3 levels and an upregulation of the mitochondrial markers PINK1, Parkin, and the LC3-II/LC3-I ratio. Leptin corrected these glutamate-induced adverse changes. In addition, the mitochondrial inhibitor, CsA, significantly reduced intracellular zinc ion content and ZnT3 expression. These results are consistent with recent findings, which showed that PINK1/parkin-mediated mitophagy was involved in ZnO NP-induced toxicity in BV-2 cells (33). In addition, the current results are partly in accordance with our previous *in vivo* study, which investigated the contribution of an autophagy inhibitor (3-methyladenine, 3-MA) on the regulation of ZnTs and the expression of the autophagy markers, LC3 and beclin-1, in the rat hippocampus following recurrent neonatal seizures. In that study, we found that the upregulated expressions of ZnT-1, ZnT-2, LC3, and beclin-1 in the recurrent seizure group at 1.5, 3, 6, and 24 h after the last seizure were remarkably attenuated by pretreatment with 3-MA. Linear correlation analysis exhibited further significant correlations between beclin1-ZnT3, beclin1-ZnT2, and LC3-ZnT-2 (34). Other evidence supporting the neuroprotective effects of leptin through autophagy signaling is derived from the finding that leptin therapy reduced cerebral ischemic injury by inhibiting the elevation of connexin 43 *in vivo* (35). Connexins have recently emerged as substrates and regulators of autophagy. Several connexin isoforms could regulate autophagy by recruiting pre-autophagosomal autophagy-related proteins to the plasma membrane (36). For instance, 6 h after TBI, p-CX43 and LC3-II expression reached a maximum level in the hippocampus of rats, while the inhibition of p-CX43 reduced the TBI-induced autophagy. This suggested a regulatory role of connexin 43 in autophagy signaling in the hippocampal neurons following TBI (37). Increased autophagy activity of osteocyte-like MLO-Y4 cells induced the internalization of connexin 43 into autophagosome/autolysosomes and was degraded by autophagy (38). It has been shown that the role of leptin in the brain appears to be mediated by the PI3K pathway (39). Zhang et al. showed that leptin attenuated cerebral ischemia injury by promoting the energy metabolism *via* PI3K/Akt (40). PI3K is a key upstream regulator of autophagy, and PI3K/Akt signaling is a well-characterized pathway that contributes to mTOR activation (41). Taken together, these studies, combined with our recent *in vivo* finding of the neuroprotective effects of leptin on cognition and the expression of ZnT3/CK-28K following “twist” developmental seizures (13), suggest that mitophagy signaling-mediated zinc dyshomeostasis might play a role in the protective mechanisms exhibited during glutamate neuroexcitoxicity. Together with elevated ZnT3 expression and mitochondrial activation, these molecules could be involved in hippocampal pathology and neuroprotection in epilepsy.

## Conclusion

This study suggests mitophagy-mediated zinc dyshomeostasis and mitochondrial activation contributed to glutamate-induced HT22 neuronal cell injury and that leptin treatment could counteract these detrimental effects. The results highlight mitophagy-mediated zinc homeostasis *via* mitochondrial activation as a potential strategy to counteract neuroexcitotoxicity.

## Author Contributions

HN was the designer and dissertation writer of this study. M-fJ was the specific operator of this experiment and was responsible for the statistical analysis of data. L-lL assisted in the completion of some experiments.

## Conflict of Interest Statement

The authors declare that the research was conducted in the absence of any commercial or financial relationships that could be construed as a potential conflict of interest. The handling editor is currently co-organizing a Research Topic with one of the authors HN and confirms the absence of any other collaboration.
